# Supplementary material for: Pre-Therapeutic VEGF Level in Plasma Is a Prognostic Bio-Marker in Head and Neck Squamous Cell Carcinoma (HNSCC)
Source: Cancers (Basel). 2021 Jul 27;13(15):3781. doi: 10.3390/cancers13153781 (PMC8345208; doi:10.3390/cancers13153781)
Supplement: Supplementary file 1 [file cancers-13-03781-s001.zip › cancers-1268840-supplementary.pdf]

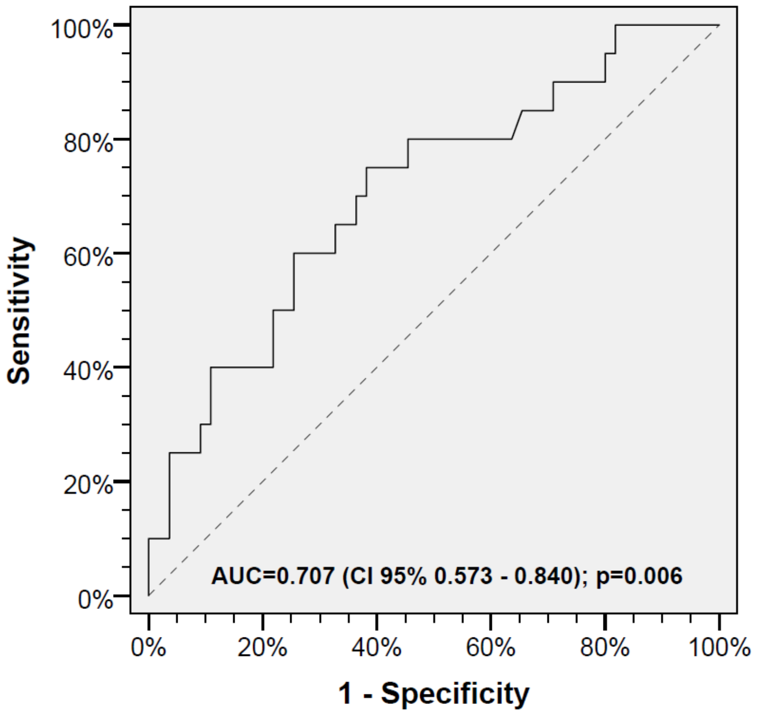

(a)

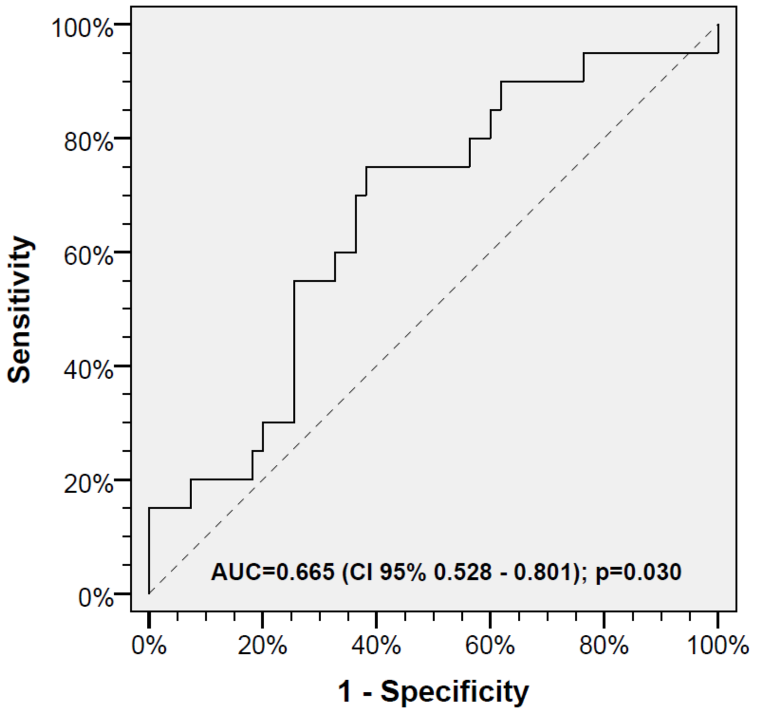

(b)

VEGF plasma (ng/L)	Sensitivity	FDR	Specificity	Youden index	Below (0) or above (1) cut-off
0	1.000	1.000	0.000	0.000	0
0.7050	1.000	0.964	0.036	0.036	0
2.2800	1.000	0.927	0.073	0.073	0
3.3650	1.000	0.909	0.091	0.091	0
3.6600	1.000	0.891	0.109	0.109	0
4.2100	1.000	0.855	0.145	0.145	0
4.6200	1.000	0.836	0.164	0.164	0
4.8950	1.000	0.818	0.182	0.182	0
5.5600	0.950	0.818	0.182	0.173	0
6.2250	0.950	0.800	0.200	0.190	0
6.8300	0.900	0.800	0.200	0.180	0
7.3950	0.900	0.782	0.218	0.196	0
7.7600	0.900	0.764	0.236	0.213	0
8.0700	0.900	0.745	0.255	0.229	0
8.2400	0.900	0.727	0.273	0.245	0
8.4050	0.900	0.709	0.291	0.262	0
8.9900	0.850	0.709	0.291	0.247	0
10.0700	0.850	0.691	0.309	0.263	0
10.7450	0.850	0.673	0.327	0.278	0
10.8900	0.850	0.655	0.345	0.294	0
11.2450	0.800	0.636	0.364	0.291	0
11.8650	0.800	0.618	0.382	0.305	0
12.9750	0.800	0.600	0.400	0.320	0
13.7900	0.800	0.582	0.418	0.335	0
14.3750	0.800	0.564	0.436	0.349	0
15.2800	0.800	0.545	0.455	0.364	0
15.6500	0.800	0.527	0.473	0.378	0
16.4700	0.800	0.509	0.491	0.393	0
17.8800	0.800	0.491	0.509	0.407	0
18.6100	0.800	0.473	0.527	0.422	0
19.0550	0.800	0.455	0.545	0.436	0
19.9300	0.750	0.455	0.545	0.409	0
20.7550	0.750	0.436	0.564	0.423	0
22.2750	0.750	0.418	0.582	0.436	0
23.9650	0.750	0.400	0.600	0.450	0
25.3700	0.750	0.382	0.618	0.464	0
26.4150	0.700	0.382	0.618	0.433	1
26.6800	0.700	0.364	0.636	0.445	1
28.0550	0.650	0.364	0.636	0.414	1
30.1900	0.650	0.345	0.655	0.425	1
31.2550	0.650	0.327	0.673	0.437	1
31.6250	0.600	0.327	0.673	0.404	1
32.0200	0.600	0.309	0.691	0.415	1
33.2800	0.600	0.291	0.709	0.425	1
36.4750	0.600	0.273	0.727	0.436	1
39.1550	0.600	0.255	0.745	0.447	1
39.7700	0.550	0.255	0.745	0.410	1
39.9300	0.500	0.255	0.745	0.373	1
40.6500	0.500	0.236	0.764	0.382	1
41.8300	0.500	0.218	0.782	0.391	1
42.4500	0.450	0.218	0.782	0.352	1
43.9650	0.400	0.218	0.782	0.313	1
45.6950	0.400	0.200	0.800	0.320	1
48.2200	0.400	0.182	0.818	0.327	1
51.2000	0.400	0.164	0.836	0.335	1
52.2950	0.400	0.145	0.855	0.342	1
53.2800	0.400	0.127	0.873	0.349	1
56.0700	0.400	0.109	0.891	0.356	1
58.8100	0.350	0.109	0.891	0.312	1
60.7100	0.300	0.109	0.891	0.267	1
62.5100	0.300	0.091	0.909	0.273	1
64.9800	0.250	0.091	0.909	0.227	1
68.3000	0.250	0.073	0.927	0.232	1
70.1550	0.250	0.055	0.945	0.236	1
75.0850	0.250	0.036	0.964	0.241	1
83.5250	0.200	0.036	0.964	0.193	1
89.9850	0.150	0.036	0.964	0.145	1
113.2250	0.100	0.036	0.964	0.096	1
136.5650	0.100	0.018	0.982	0.098	1
144.0100	0.100	0.000	1.000	0.100	1
177.6450	0.050	0.000	1.000	0.050	1
206.3200	0.000	0.000	1.000	0.000	1

(c)

VEGF serum (ng/L)	Sensitivity	FDR	Specificity	Youden index	Below (0) or above (1) cut-off
0	1.000	1.000	0.000	0.000	0
9.7800	0.950	1.000	0.000	0.000	0
23.0950	0.950	0.982	0.018	0.017	0
31.9700	0.950	0.964	0.036	0.035	0
43.0950	0.950	0.945	0.055	0.052	0
51.5400	0.950	0.927	0.073	0.069	0
54.5250	0.950	0.909	0.091	0.086	0
56.8150	0.950	0.891	0.109	0.104	0
59.4500	0.950	0.873	0.127	0.121	0
61.5350	0.950	0.855	0.145	0.138	0
63.6200	0.950	0.836	0.164	0.155	0
68.7050	0.950	0.818	0.182	0.173	0
72.5900	0.950	0.800	0.200	0.190	0
74.6900	0.950	0.782	0.218	0.207	0
80.8600	0.950	0.764	0.236	0.225	0
86.1700	0.900	0.764	0.236	0.213	0
93.9750	0.900	0.745	0.255	0.229	0
101.1100	0.900	0.727	0.273	0.245	0
106.5750	0.900	0.709	0.291	0.262	0
113.8200	0.900	0.691	0.309	0.278	0
116.6850	0.900	0.673	0.327	0.295	0
117.5700	0.900	0.655	0.345	0.311	0
119.0450	0.900	0.636	0.364	0.327	0
120.8250	0.900	0.618	0.382	0.344	0
123.5800	0.850	0.618	0.382	0.325	0
127.3400	0.850	0.600	0.400	0.340	0
132.6800	0.800	0.600	0.400	0.320	0
137.0600	0.800	0.582	0.418	0.335	0
140.7150	0.800	0.564	0.436	0.349	0
144.5850	0.750	0.564	0.436	0.327	0
146.7000	0.750	0.545	0.455	0.341	0
148.9850	0.750	0.527	0.473	0.355	0
150.5250	0.750	0.509	0.491	0.368	0
158.3050	0.750	0.491	0.509	0.382	0
166.5000	0.750	0.473	0.527	0.395	0
167.8050	0.750	0.455	0.545	0.409	0
171.0450	0.750	0.436	0.564	0.423	0
176.0900	0.750	0.418	0.582	0.436	0
179.7500	0.750	0.400	0.600	0.450	0
182.9600	0.750	0.382	0.618	0.464	0
187.0150	0.700	0.382	0.618	0.433	0
193.0300	0.700	0.364	0.636	0.445	0
199.8100	0.650	0.364	0.636	0.414	0
208.0700	0.600	0.364	0.636	0.382	0
221.5650	0.600	0.345	0.655	0.393	0
233.3850	0.600	0.327	0.673	0.404	0
236.6700	0.550	0.327	0.673	0.370	0
239.4400	0.550	0.309	0.691	0.380	0
246.4950	0.550	0.291	0.709	0.390	0
251.0900	0.550	0.273	0.727	0.400	0
263.6550	0.550	0.255	0.745	0.410	0
264.6000	0.500	0.255	0.745	0.373	1
265.6450	0.450	0.255	0.745	0.335	1
266.5000	0.400	0.255	0.745	0.298	1
267.5550	0.350	0.255	0.745	0.261	1
275.6050	0.300	0.255	0.745	0.224	1
284.7700	0.300	0.236	0.764	0.229	1
292.0100	0.300	0.218	0.782	0.235	1
298.1000	0.300	0.200	0.800	0.240	1
306.2550	0.250	0.200	0.800	0.200	1
322.3750	0.250	0.182	0.818	0.205	1
332.0100	0.200	0.182	0.818	0.164	1
334.3550	0.200	0.164	0.836	0.167	1
344.7850	0.200	0.145	0.855	0.171	1
354.2600	0.200	0.127	0.873	0.175	1
358.8300	0.200	0.109	0.891	0.178	1
365.8950	0.200	0.091	0.909	0.182	1
397.2250	0.200	0.073	0.927	0.185	1
442.7150	0.150	0.073	0.927	0.139	1
466.4500	0.150	0.055	0.945	0.142	1
473.6150	0.150	0.036	0.964	0.145	1
481.2500	0.150	0.018	0.982	0.147	1
494.5850	0.150	0.000	1.000	0.150	1
593.5100	0.100	0.000	1.000	0.100	1
756.3450	0.050	0.000	1.000	0.050	1
826.3500	0.000	0.000	1.000	0.000	1

(d)

Figure S1. Receiver-operator characteristics (ROC) of vascular endothelial growth factor (VEGF) concentration in plasma [(a), (c)] and serum [(b), (d)] for event-free survival of therapy-naïve head and neck squamous cell carcinoma patients. (a) ROC for $VEGF_{Plasma}$; (b) ROC for $VEGF_{Serum}$; (c) tabulated sensitivity, false-discovery rate (FDR), specificity, and Youden indices for $VEGF_{Plasma}$ to define the cut-off for $VEGF_{Plasma}$ of 26 ng/L according the maximum Youden score (underlined); (d) tabulated sensitivity, false-discovery rate (FDR), specificity, and Youden indices for $VEGF_{Serum}$ to define the cut-off for $VEGF_{Serum}$ of 264 ng/L according the maximum Youden score (underlined).

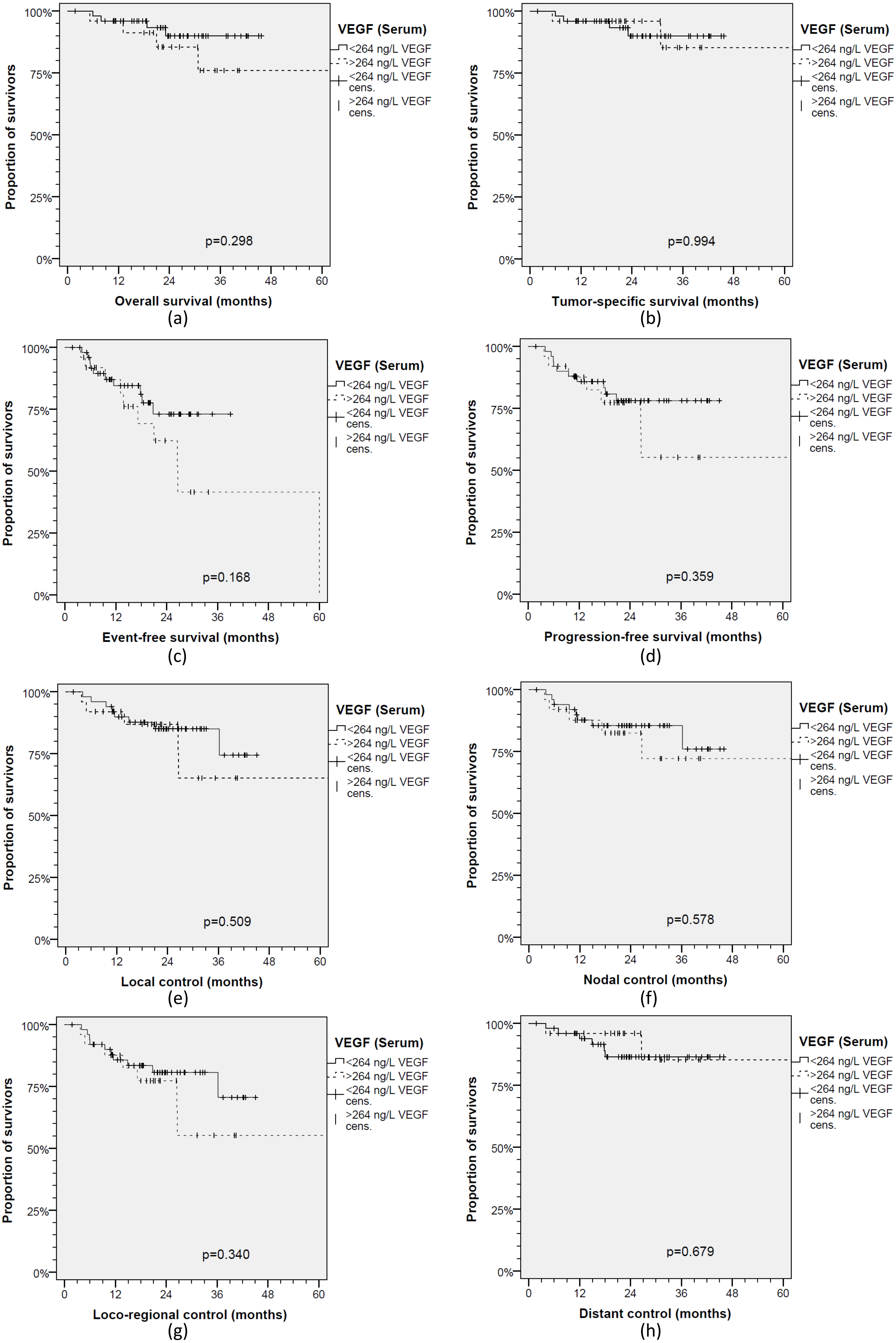

Figure S2. Kaplan-Meier plots for cumulative survival in head and neck squamous cell carcinoma patients from the test cohort stratified according to vascular endothelial growth factor (VEGF) in pre-therapeutic serum. (a) overall survival; (b) tumor-specific survival; (c) event-free survival; (d) progression-free survival; (e) local control; (f) nodal control; (g) loco-regional control; (h) distant control. *p* values shown are from 2-sided log-rank test.
